# Genetic Reduction of the Translational Repressors FMRP and 4E‐BP2 Preserves Memory in Mouse Models of Alzheimer's Disease

**DOI:** 10.1111/acel.70315

**Published:** 2025-12-03

**Authors:** Felipe C. Ribeiro, Danielle Cozachenco, Michael Parkhill, Brandon Rodrigue, Caio Borges, Jean‐Claude Lacaille, Karim Nader, Fernanda G. De Felice, Mychael V. Lourenco, Argel Aguilar‐Valles, Nahum Sonenberg, Sergio T. Ferreira

**Affiliations:** ^1^ Institute of Medical Biochemistry Leopoldo de Meis Federal University of Rio de Janeiro Rio de Janeiro Brazil; ^2^ Department of Neuroscience Carleton University Ottawa Ontario Canada; ^3^ Department of Neurosciences, Centre for Interdisciplinary Research on Brain and Learning and Research Group on Neural Signaling and Circuits Université de Montréal Montreal Quebec Canada; ^4^ Department of Psychology McGill University Montreal Quebec Canada; ^5^ Department of Biomedical and Molecular Sciences, Centre for Neuroscience Studies Queen's University Kingston Ontario Canada; ^6^ Department of Psychiatry Queen's University Kingston Ontario Canada; ^7^ D'or Institute for Research and Education Rio de Janeiro Brazil; ^8^ Department of Biochemistry and Tissue Biology State University of Campinas Campinas Brazil; ^9^ Department of Biochemistry McGill University Montreal Quebec Canada; ^10^ Institute of Biophysics Carlos Chagas Filho Federal University of Rio de Janeiro Rio de Janeiro Brazil

**Keywords:** 4E‐BP2, Alzheimer's disease, fragile X messenger ribonucleoprotein, memory, mRNA translation

## Abstract

Alzheimer's disease (AD) is characterized by progressive memory decline. Converging evidence indicates that hippocampal mRNA translation (protein synthesis) is defective in AD. Here, we show that genetic reduction of the translational repressors, Fragile X messenger ribonucleoprotein (FMRP) or eukaryotic initiation factor 4E (eIF4E)‐binding protein 2 (4E‐BP2), prevented the attenuation of hippocampal protein synthesis and memory impairment induced by AD‐linked amyloid‐β oligomers (AβOs) in mice. Moreover, genetic reduction of 4E‐BP2 rescued memory deficits in aged APPswe/PS1dE9 (APP/PS1) transgenic mouse model of AD. Our findings demonstrate that strategies targeting repressors of mRNA translation correct hippocampal protein synthesis and memory deficits in AD models. Results suggest that modulating pathways controlling brain mRNA translation may confer memory benefits in AD.

## Graphical abstract

Genetic reduction of the translational repressors FMRP or 4E‐BP2 prevents amyloid‐β oligomer–induced suppression of hippocampal protein synthesis and memory loss in mice. Targeting translational control preserves cognitive function in Alzheimer's models, revealing mRNA translation as a promising therapeutic axis for memory preservation. Created in BioRender. Lourenco, M. (2025) https://BioRender.com/xsz031c.

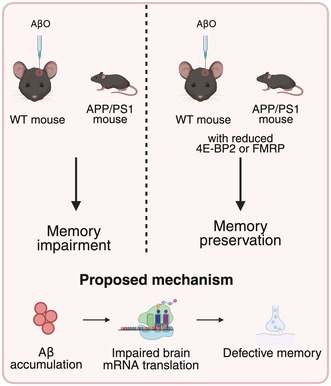

**Abbreviations**
4E‐BP2: eukaryotic initiation factor 4E (eIF4E)‐binding protein 2
AD: Alzheimer's disease
AβOs: amyloid‐β oligomers
CYFIP1: cytoplasmic FMR1‐interacting protein 1
eEF2: eukaryotic elongation factor 2
eIF2α: eukaryotic translation initiation factor 2α
eIF4E: eukaryotic translation initiation factor 4E
FMRP: Fragile X messenger ribonucleoprotein
ISR: integrated stress response
ISRIB: integrated stress response inhibitor
SUnSET: surface sensing of translation
WT: wild type

Alzheimer's disease (AD) is the most prevalent cause of dementia in the elderly, and is characterized by progressive cognitive decline (Ferreira et al. 2015). Hippocampal mRNA translation (protein synthesis) is crucial for memory consolidation by promoting synapse strengthening (Costa‐Mattioli et al. 2009). Signaling pathways controlling the initiation and elongation steps of mRNA translation are deregulated in the brains of AD patients and mouse models (Oliveira et al. 2021). This results in attenuation of global brain protein synthesis, changes in brain proteome composition (Elder et al. 2021), and memory failure in AD mouse models (Oliveira et al. 2021; Elder et al. 2021; Lourenco et al. 2013). These findings suggest that multiple pathways converge to arrest hippocampal mRNA translation, thereby contributing to AD pathogenesis.

Fragile X messenger ribonucleoprotein (FMRP, encoded by the *Fmr1* gene) is a negative regulator of protein synthesis that blocks both the initiation and elongation phases of mRNA translation (Darnell and Klann 2013). However, the roles of FMRP in AD have not been fully understood. Eukaryotic initiation factor 4E‐binding protein 2 (4E‐BP2, encoded by the *Eif4ebp2* gene) is the major 4E‐BP isoform in the brain (Banko et al. 2005). 4E‐BP2 is a translational repressor that binds to eIF4E and prevents the formation of the eIF4F complex (Banko et al. 2005). Here, we tested the hypothesis that genetic reduction of FMRP or 4E‐BP2 could rescue memory deficits in AD mouse models.

We initially investigated whether genetic reduction of FMRP or 4E‐BP2 prevented the attenuation of hippocampal protein synthesis induced by amyloid‐β oligomers (AβOs), soluble neurotoxins that accumulate in AD brains and cause synapse failure and cognitive impairment (Ferreira et al. 2015; Lourenco et al. 2013). We infused 10 pmol AβOs (or vehicle) via intracerebroventricular (i.c.v.) injections in wild type (WT), *Fmr1* ^y/−^ or *Eif4ebp2* ^+/−^ mice, and assessed global *de novo* protein synthesis using surface sensing of translation (SUnSET) in ex vivo hippocampal slices. In line with our previous reports (Oliveira et al. 2021; Banko et al. 2005; Napoli et al. 2008; Ribeiro et al. 2023; Ribeiro et al. 2024), we found that i.c.v. infusion of AβOs caused a reduction of approximately 30% in *de novo* protein synthesis in hippocampal slices obtained from WT mice (Figure 1A,B and Data S1). In contrast, infusion of AβOs did not attenuate hippocampal protein synthesis (compared to vehicle‐infused mice) in either *Fmr1* ^y/−^ or *Eif4ebp2* ^+/−^ mice (Figure 1A,B).

**FIGURE 1:**
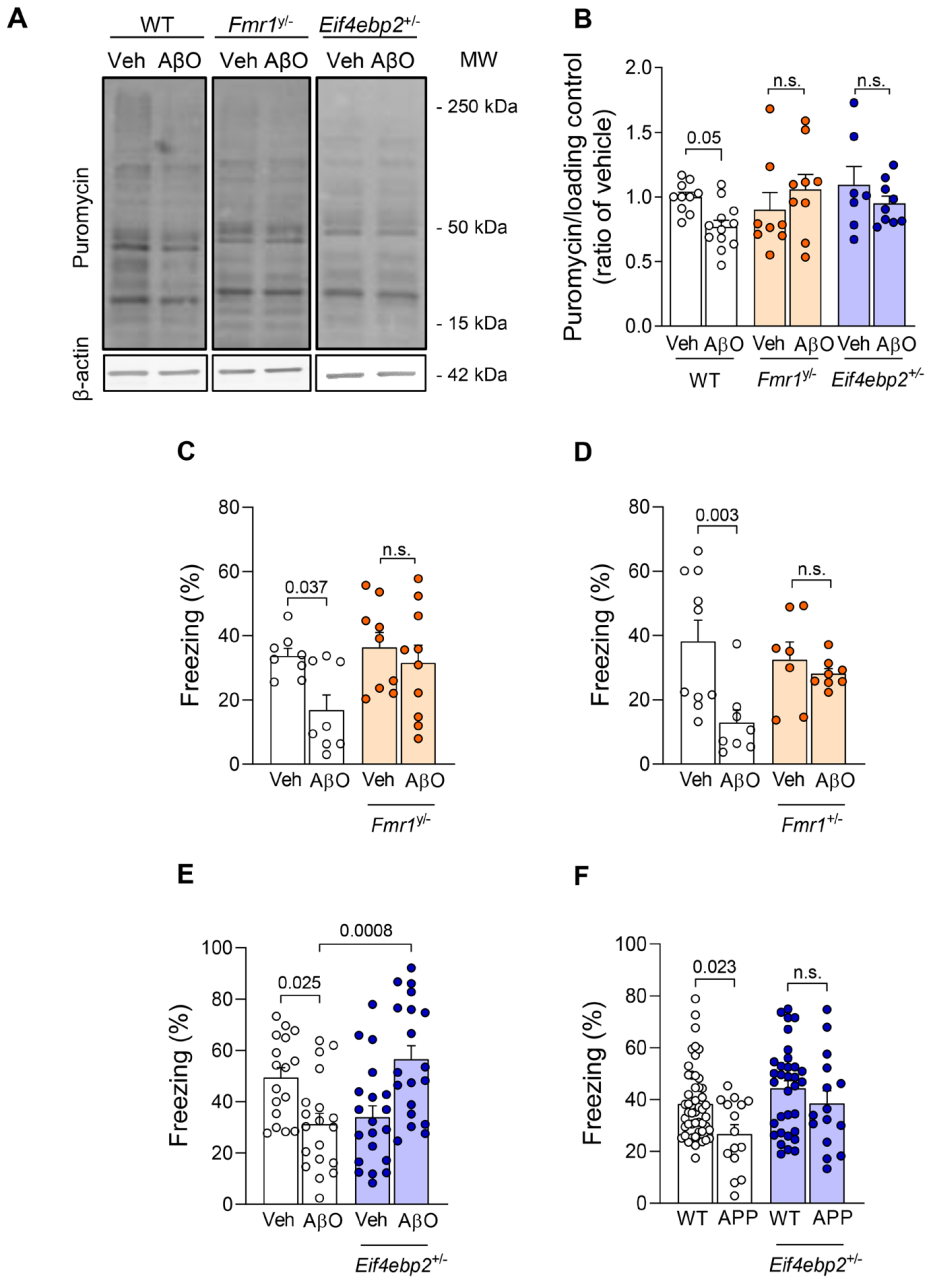
Genetic reduction of FMRP or eIF4E‐BP2 rescues defective hippocampal protein synthesis and memory in AβO‐infused mice. (A, B) 3‐month‐old male *Eif4ebp2* ^+/−^ or *Fmr1* ^y/−^ mice (or corresponding WT littermates) received an i.c.v. infusion of 10 pmol AβOs (or vehicle) (*N* = 10 Veh, 12 AβOs, 8 Fmr1^y/−^ Veh, 9 Fmr1^y/−^ AβOs, 7 Eif4ebp2^+/−^ Veh, 9 Eif4ebp2^+/−^ AβOs). Hippocampal slices were obtained 7 days later and were exposed to puromycin (5 μg/mL; 45 min) for SUnSET analysis of *de novo* protein synthesis. Puromycin levels were evaluated by Western blotting. (C–E) 3‐month‐old *Fmr1* ^y/−^ (*N* = 8 Veh, 9 AβOs, 9 Fmr1^/−^ Veh, 10 Fmr1^y/−^ AβOs), *Fmr1* ^+/−^ (*N* = 10 Veh, 8 AβOs, 7 *Fmr1* ^+/−^ Veh, 8 *Fmr1* ^+/−^ AβOs) or *Eif4ebp2* ^+/−^ mice (*N* = 17 Veh, 19 AβOs, 20 *Eif4ebp2* ^+/−^ Veh, 19 *Eif4ebp2* ^+/−^ AβOs) (or corresponding WT littermates) received an i.c.v. infusion of 10 pmol AβOs (or vehicle) and memory was assessed using the contextual fear conditioning (CFC) test 7 days later. (F) 12‐month‐old APPswe/PS1dE9/*Eif4ebp2* ^+/−^ mice and corresponding controls (APPswe/PS1DE9/*Eif4ebp2* ^*+/+*^ mice) were assessed in the CFC memory test (*N* = 49 WT, 15 APP/PS1, 33 WT *Eif4ebp2* ^*+/−*^, 15 APP/PS1 *Eif4ebp2* ^*+/−*^); *p*‐values are shown for appropriate comparisons; Two‐way ANOVA followed by Holm‐Sidak post hoc test.

We next assessed the impact of AβOs on hippocampus‐dependent fear memory using a contextual fear conditioning paradigm in *Fmr1* ^y/−^ males, *Fmr1* ^+/−^ females or WT mice. As expected, i.c.v. infusion of AβOs blocked long‐term fear memory in WT mice (Figure 1C,D). Remarkably, *Fmr1* ^y/−^ or *Fmr1* ^*+/−*^ mice that received infusions of AβOs exhibited intact memory in the fear conditioning test (Figure 1C,D).

Next, *Eif4ebp2* ^+/−^ or WT mice were tested in the contextual fear conditioning paradigm after i.c.v. infusion of 10 pmol AβOs (or vehicle). Consistent with the notion that translational control by 4E‐BP2 plays an essential role in memory processes and must be tightly regulated (Banko et al. 2005), 4E‐BP2 haploinsufficiency per se led to memory impairment in vehicle‐infused mice (Figure 1E). Interestingly, however, 4E‐BP2 haploinsufficiency prevented AβO‐induced impairment of fear memory (Figure 1E). Control measurements showed that genetic reduction of *Fmr1* or *Eif4ebp2* had no impact on the locomotor activity of mice (not shown). Altogether, these findings indicate that alleviating translational repression via genetic reduction of 4E‐BP2 or FMRP prevents AβO‐induced memory impairments.

To determine whether alleviating translational repression could represent an effective approach to rescue memory in a chronic AD model, we generated APPswe/PS1dE9 (APP/PS1) mice harboring a single 4E‐BP2 allele (*APP/PS1/4E‐BP2* ^*+/−*^ mice). APP/PS1 mice develop age‐related Aβ accumulation and memory impairment (Ribeiro et al. 2024; Lourenco et al. 2019). Whereas aged APP/PS1 mice (harboring two copies of the *4E‐BP2* allele) exhibited impaired long‐term fear memory, *4E‐BP2* haploinsufficient APP/PS1 mice exhibited memory performance comparable to that of wild type (WT) mice (Figure 1F). Control experiments revealed that hippocampal 4E‐BP2 and FMRP levels were not altered in APP/PS1 mice compared to WT littermates (Figure S1).

Our results demonstrate that genetic brake‐release of hippocampal mRNA translation preserves and rescues memory in acute and chronic mouse models of AD, respectively. The findings using mice with genetically reduced 4E‐BP2 or FMRP support the concept that correcting brain protein synthesis defects counteracts AD‐linked memory impairment. Interestingly, we did not observe significant differences in baseline hippocampal protein synthesis in *Fmr1* ^y/−^ or *Eif4ebp2* ^+/−^ mice compared to WT mice. Thus, while genetic reduction of FMRP or 4E‐BP2 did not appear to have a major impact on global hippocampal protein synthesis, it was sufficient to block the attenuation of protein synthesis induced by AβOs. Changes in the brain proteome of *Fmr1* KO mice have been extensively investigated, including changes in activity‐dependent synaptic proteins. Of note, FMRP was shown to control mGluR5‐induced translation of App mRNA (Westmark and Malter 2007), which may have important implications for brain amyloid‐β accumulation. 4E‐BP2 was also shown to control the translation of specific mRNAs such as neuroligins and AMPA receptor subunits, which are important for synapses and memory (Aguilar‐Valles et al. 2018). Therefore, derepression of specific mRNA subsets, notably synapse‐related ones, may underlie the protective effects of translational brake release in AD models.

Both 4E‐BP2 and FMRP appear to regulate the translation of specific mRNA subsets. For example, constitutive KO of 4E‐BP2 specifically affects cognitive aspects dependent on the hippocampus, but not on the amygdala (Banko et al. 2005; Banko et al. 2007; Banko et al. 2006). Thus, it is conceivable that there is a specific impact of the genetic reduction of FMRP or 4E‐BP2 in the translation of proteins involved in either the development of the hippocampus or in transmission and plasticity, which may explain the targeting of cognitive processes that become impaired in AD models. Further studies thus appear warranted to elucidate the specific hippocampal mRNAs that are regulated by translational repressors and the detailed mechanisms by which genetic reduction of *Fmr1* and *4E‐BP* protects brain function in AD.

In conclusion, while we and others have previously demonstrated that defective proteostasis regulation (Ribeiro et al. 2023; Duran‐Aniotz et al. 2023) and translational control (Oliveira et al. 2021; Lourenco et al. 2013; Ma et al. 2013) play important roles in AD, the current study underscores the importance of inhibiting two key regulators of translation in Aβ‐induced memory impairments. While basal levels of 4E‐BP2 and FMRP were not altered in APP/PS1 mice (Figure S1), our findings warrant future mechanistic investigation into how these multiple dysregulated pathways converge to trigger AD‐linked symptoms. The notion that control of mRNA translation may be a druggable target may pave new roads toward effective therapeutics in AD.

## 1 Experimental Procedures

### 1.1 Animals

Adult male or female mice (P90) were obtained from the animal facility at McGill University and were housed in groups of 2–5 mice per cage, with food and water *ad libitum*, on a 12 h light/dark cycle. Male and female APPswe/PS1dE9 mice (Jackson Laboratories, strain #005864) (Ribeiro et al. 2024), *Eif4ebp2* ^+/−^ (Jackson Laboratories, strain #031507) (Aguilar‐Valles et al. 2021), *Fmr1* ^+/−^ and *Fmr1* ^y/−^ (Jackson Laboratories, strain #003025) (Gantois et al. 2017) mice on a C57BL/6J background (obtained from The Jackson Laboratories) were bred at our facilities. APP/PS1 mice were obtained from JAX and used 3 generations after initial breeding in the Sonenberg lab. APP/PS1/4E‐BP2^+/−^ crossed mice were used as F1 (with all possible resulting genotypes). 4E‐BP2^+/−^ mice were around 24 generations in the Sonenberg lab. *Fmr1* ^*+/−*^ mice were around 6 generations. Breeders were renewed every 6 months. Experiments involving APP/PS1 mice were performed in 12‐month‐old animals. Experiments involving AβO‐injected mice were performed in 3‐month‐old mice. All procedures followed the Canadian Council on Animal Care guidelines and were approved by McGill University Committees.

### 1.2 AβO Preparation and Intracerebroventricular (i.c.v.) Infusion

Oligomerization of the Aβ_1‐42_ peptide (#641–15, California Peptide, Salt Lake, CA) and intracerebroventricular (i.c.v.) infusion of 10 pmol AβOs (or vehicle) in mice were performed as described (Lourenco et al. 2013; Figueiredo et al. 2013). Previous studies from our group have shown that i.c.v. infusion of AβOs has no noticeable impact on locomotor behavior in mice, i.e., distance traveled/average velocity (Oliveira et al. 2021) or crossings (Ledo et al. 2013).

### 1.3 Surface Sensing of Translation (SUnSET) in Hippocampal Slices

Hippocampal slices (400 μm) were obtained and kept in artificial cerebrospinal fluid (aCSF; 124 mM NaCl, 4.4 mM KCl, 1 mM Na_2_HPO_4_, 25 mM NaHCO_3_, 2 mM MgCl_2_, 2 mM CaCl_2_, 10 mM glucose) for 2 h. Newly synthesized polypeptides were detected using SUnSET as described (Oliveira et al. 2021). Hippocampal slices were prepared 7 days after i.c.v. infusions of 10 pmol AβOs (or vehicle) in *Eif4ebp2* ^+/−^, *Fmr1* ^y/−^ or corresponding littermate WT mice. Slices were exposed to puromycin (5 μg/mL; 45 min) and collected for Western blotting. All membranes used for quantification are presented in Data S1.

### 1.4 Western Blotting

Hippocampal slices were dissociated using the Bio‐Plex Cell Lysis Kit (Bio‐Rad), centrifuged for 10 min at 10,000 g at 4°C, and the supernatant was collected (Ribeiro et al. 2023). Protein concentration was determined using the Bradford Protein Assay Kit. Fifty μg total protein were resolved in 12% Tris‐glycine SDS‐PAGE gels, transferred to nitrocellulose membranes, and incubated with anti‐puromycin (12D10 clone; #MABE343; 1:1000; EMD Millipore) and anti‐β‐actin (#ab170325) or anti‐β‐tubulin (#ab15568) used as loading controls (1:10,000; Abcam). Immunoblots were developed using IR dye‐conjugated fluorescent secondary antibodies (#926–32,210 or #926–32,211; 1:5000; LiCor), imaged on an Odyssey system, and analyzed using ImageJ (NIH).

### 1.5 Contextual Fear Conditioning

In the training phase, mice were placed in the apparatus and received two foot shocks (0.35 mA for 1 s at 2 min and 2:30 min) and were placed back in their home cages 30 s later. For the test phase (24 h later), mice were placed in the same apparatus for 5 min, with no shock. Freezing behavior was recorded automatically on Freeze Frame (Harvard Apparatus, Holliston, MA).

### 1.6 Statistical Analysis

Data are expressed as means ± S.E.M. and were analyzed on GraphPad Prism 8 (La Jolla, CA) using two‐tailed two‐way ANOVA followed by Holm‐Sidak post‐test. Sample sizes and *p*‐values for each experiment are indicated in the figure legend.

## Author Contributions

F.C.R., D.C., A.A.‐V. and S.T.F. designed the study. F.C.R., D.C., M.P., B.R., C.B. and A.A.‐V. performed research. F.C.R., D.C., C.B. and A.A.‐.V. analyzed data. K.N., M.V.L., J.‐C.L., F.G.D.F., A.A.‐V., N.S. and S.T.F. contributed reagents, materials, animals and analysis tools. F.C.R., D.C., F.G.D.F., M.V.L., A.A.‐V., N.S. and S.T.F. analyzed and discussed results. F.C.R., D.C., M.V.L. and S.T.F. wrote the manuscript with inputs from the other authors.

## Funding

This work was supported by grants from Fundação Carlos Chagas Filho de Amparo à Pesquisa do Estado do Rio de Janeiro (FAPERJ) (STF, FGDF and MVL), Conselho Nacional de Desenvolvimento Científico e Tecnológico (CNPq) (STF, FGDF and MVL), National Institute of Translational Neuroscience (INNT/Brazil) (STF and FGDF), Instituto Nacional de Ciência e Tecnologia Saúde Cerebral (INSC 406020/2022‐1/CNPq, Brazil to MVL), Alzheimer's Society Canada (NS and STF), International Brain Research Organization (MVL and AAV), Alzheimer's Association (AARF‐21‐848798 to FCR; AARG‐D‐615714 to MVL), Serrapilheira Institute (R‐2012‐37967 to MVL), and by travel grants from International Society for Neurochemistry (ISN), International Union of Biochemistry and Molecular Biology (IUBMB), American Society for Biochemistry and Molecular Biology (ASBMB) and Company of Biologists (to FCR). JCL holds the Canada Research Chair in Cellular and Molecular Neurophysiology (CRC 950‐231066), and FGDF holds the Canada Research Chair in Brain Resilience (CRC‐2023‐00155).

## Conflicts of Interest

The authors declare no conflicts of interest.

## Supporting information


**Figure S1:** Levels of FMRP and 4E‐BP2 are unchanged in the APP/PS1 hippocampus. (A) Representative blots of FMRP and 4E‐BP2 in APP/PS1 or corresponding WT littermates. (B and C) Quantification of FMRP (B) and 4E BP2 (C) in APP/PS1 hippocampi (*N* = 7 per group; Unpaired Student's *t*‐test; *p* values indicated in the panels). β‐actin was used as a loading control.

## Data Availability

The data that support the findings of this study are available on request from the corresponding author. The data are not publicly available due to privacy or ethical restrictions.
