# Supplementary material for: Genetic Reduction of the Translational Repressors FMRP and 4E‐BP2 Preserves Memory in Mouse Models of Alzheimer's Disease
Source: Aging Cell. 2025 Dec 3;25(1):e70315. doi: 10.1111/acel.70315 (PMC12741208; doi:10.1111/acel.70315)
Supplement: Supplementary file 1 — Figure S1: Levels of FMRP and 4E‐BP2 are unchanged in the APP/PS1 hippocampus. (A) Representative blots of FMRP and 4E‐BP2 in APP/PS1 or corresponding WT littermates. (B and C) Quantification of FMRP (B) and 4E BP2 (C) in APP/PS1 hippocampi (N = 7 per group; Unpaired Student's t‐test; p values indicated in the panels). β‐actin was used as a loading control. [file ACEL-25-e70315-s001.pdf]

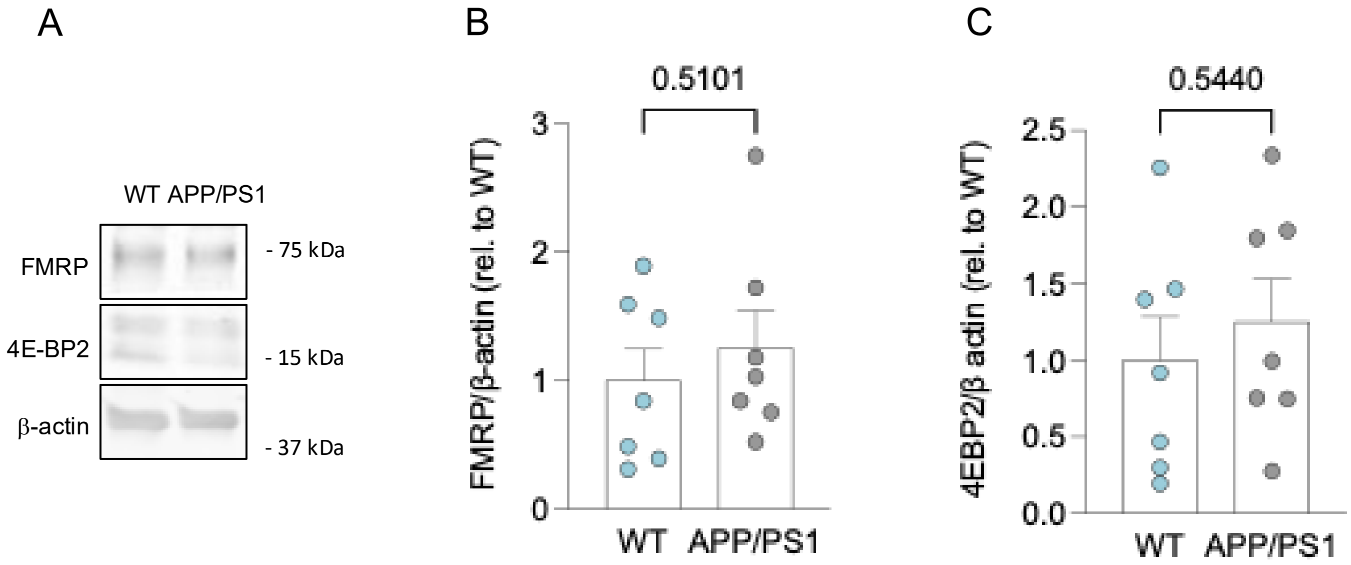

Figure S1. Levels of FMRP and 4E-BP2 are unchanged in the APP/PS1 hippocampus. (A) Representative blots of FMRP and 4E-BP2 in APP/PS1 or corresponding WT littermates. (B and C) Quantification of FMRP (B) and 4E-BP2 (C) in APP/PS1 hippocampi (N = 7 per group; Unpaired Student's t-test; p values indicated in the panels). β-actin was used as a loading control.

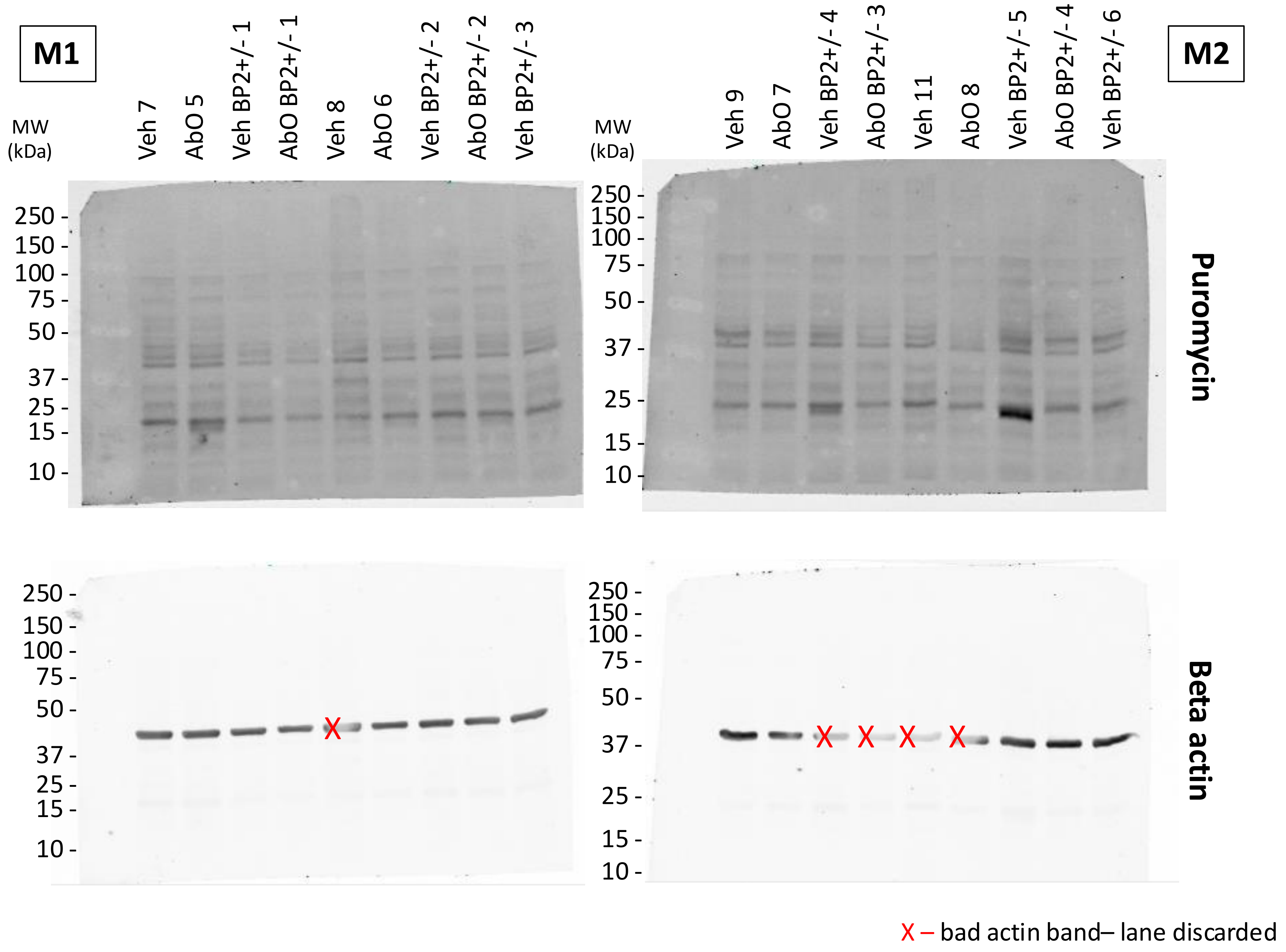

M1
M2
MW (kDa)
Veh 7
AbO 5
Veh BP2+/- 1
AbO BP2+/- 1
Veh 8
AbO 6
Veh BP2+/- 2
AbO BP2+/- 2
Veh BP2+/- 3
Veh 9
AbO 7
Veh BP2+/- 4
AbO BP2+/- 3
Veh 11
AbO 8
Veh BP2+/- 5
AbO BP2+/- 4
Veh BP2+/- 6
250
150
100
75
50
37
25
15
10
Puromycin
Beta actin
X – bad actin band– lane discarded

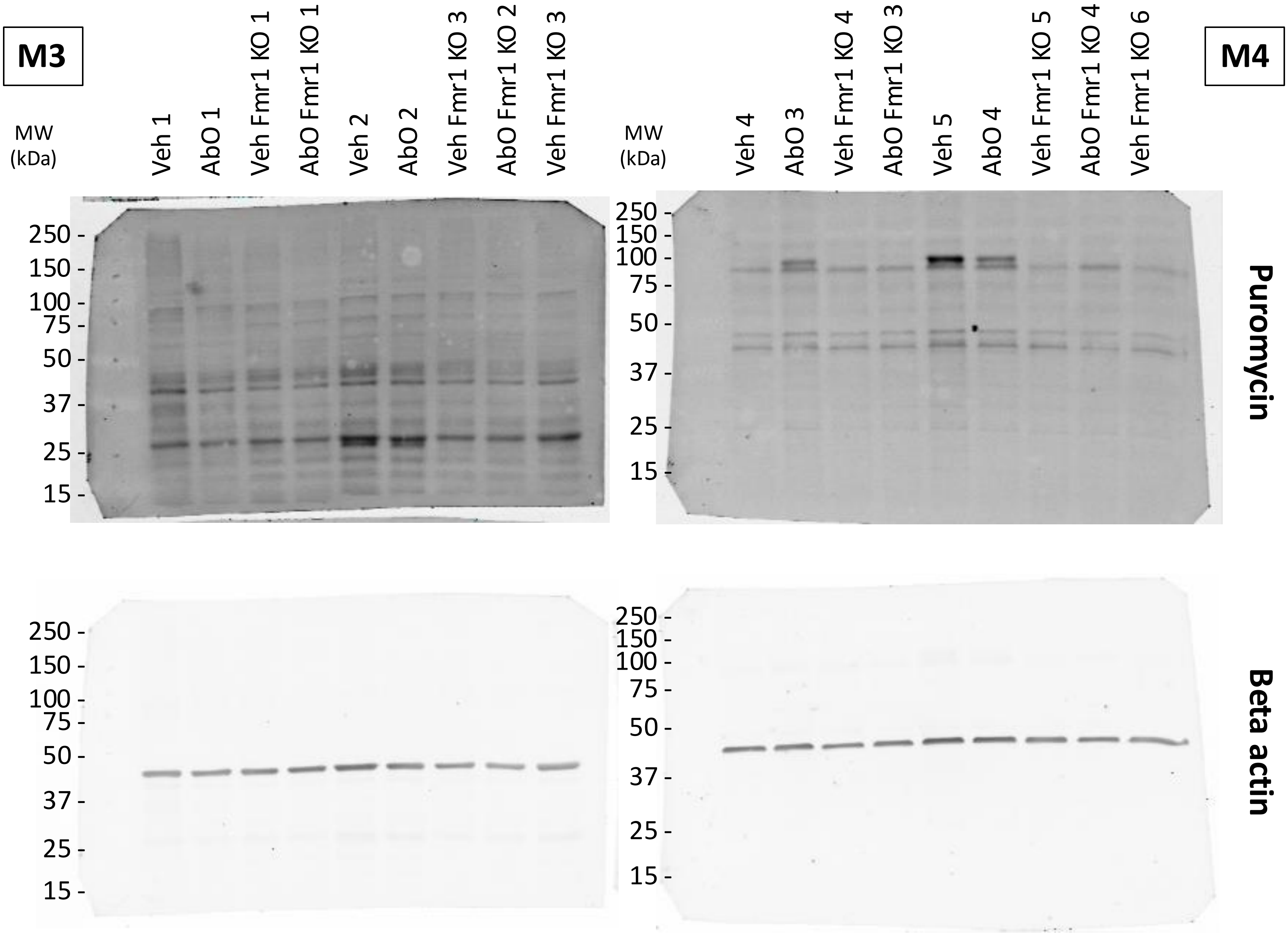

M3
M4
MW (kDa)
Veh 1
AbO 1
Veh Fmr1 KO 1
AbO Fmr1 KO 1
Veh 2
AbO 2
Veh Fmr1 KO 3
AbO Fmr1 KO 2
Veh Fmr1 KO 3
Veh 4
AbO 3
Veh Fmr1 KO 4
AbO Fmr1 KO 3
Veh 5
AbO 4
Veh Fmr1 KO 5
AbO Fmr1 KO 4
Veh Fmr1 KO 6
250
150
100
75
50
37
25
15
Puromycin
Beta actin

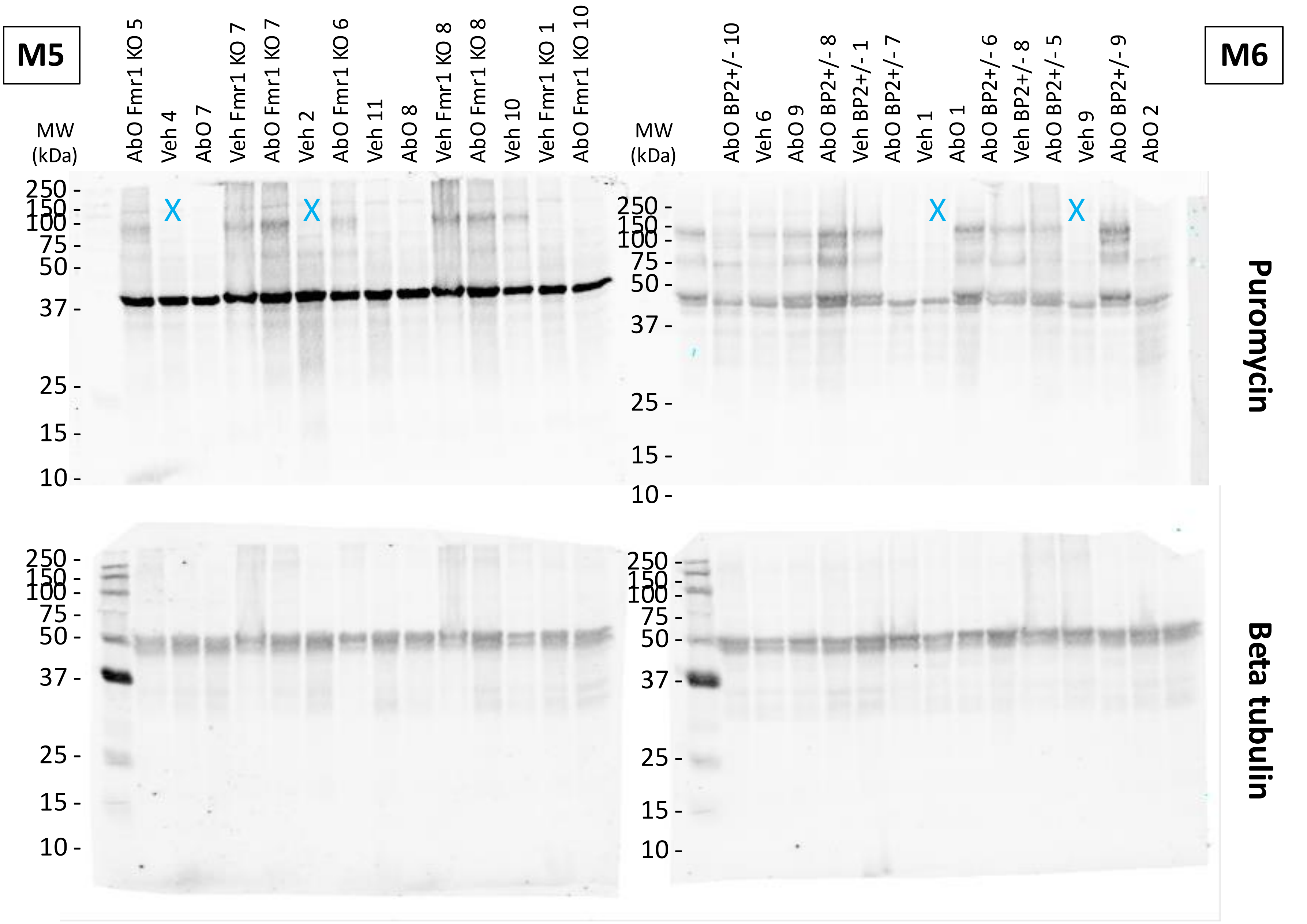

X – sample repeated from other membranes; not used for quantification

Supporting Information: Raw western blotting images used for the SUnSET quantification. The quantification was performed of the labeled smear in the entire lane. Samples marked in red X in M1 and M2 were excluded due to weak β-actin labeling. Samples marked in blue X in M5 and M6 were not used in the analysis, as they corresponded to duplicates of samples that had been run in previous gels and had already been included in the analysis.
